# Assessment of dimensional accuracy, fracture toughness, biaxial flexural strength, and surface roughness of nanozeolite reinforced 3D-printed denture base resin (In-vitro study)

**DOI:** 10.1186/s12903-025-06142-8

**Published:** 2025-05-21

**Authors:** Pansai A. Mohamed, Omnia Ghabour, Yomna Ibrahim, Mai M. Eldokmak

**Affiliations:** 1 Dental Biomaterials Department, Faculty of Dentistry, Alexandria University, Champollion Street – Azarita, Alexandria, 21521 Egypt; 2 Department of Dental Biomaterials, College of Dentistry, Arab Academy for Science, Technology and Maritime Transport (AASTMT), El-Alamein, Egypt

**Keywords:** 3D-printing, Denture base resins, Nanozeolite, Thermal cycling, Weibull analysis

## Abstract

**Background:**

3D-printed denture base resins have limited mechanical properties; therefore, several attempts were made to improve such properties. The aim of this study was to evaluate the effect of modifying a 3D-printed denture base resin with nanozeolite particles on dimensional accuracy (DA), fracture toughness (FT), biaxial flexural strength (BFS), and surface roughness (SR).

**Methods:**

Nanozeolite particles were added to 3D-printed methacrylate-based denture base resin to produce the following groups: Control, 0.25% nanozeolite, and 0.5% nanozeolite. Dimensional accuracy was assessed with a digital calliper. Fracture toughness was assessed by the single edge notched beam method (SENB) followed by Weibull analysis and work of failure. Biaxial flexural strength was tested with a universal testing machine and surface roughness was assessed with a contact profilometer. FT, BFS, and SR were assessed before and after thermal cycling of 600 cycles. Two-way ANOVA test followed by Tukey post hoc test were conducted for FT, BFS, and SR. Kruskal–Wallis test was used to compare the percent error in length, width, thickness, and percent change in FT, BFS, and surface roughness among groups with subsequent Dunn post hoc test with Bonferroni correction (α = 0.05).

**Results:**

The printing DA results revealed that the control had the highest percent error in length and width with no significant difference among the study groups, whereas the 0.5% nanozeolite group presented the highest percent error in thickness with a significant difference compared to the control. The results of FT displayed a significant statistical interaction between the resin filler content and thermal cycling (*P* = 0.001). BFS was significantly affected by the nanozeolite filler content (*P* < 0.001) with the 0.25% nanozeolite group displaying the highest mean values before and after thermal cycling. The SR results revealed a statistically significant interaction between the filler content and thermal aging (*P* < 0.001). The 0.5% nanozeolite group displayed the lowest SR mean values before and after thermal aging.

**Conclusions:**

The addition of nanozeolite enhanced the FT, BFS, and SR, however, care should be given to the optimum percentage added to the resin to attain optimum properties which would enhance the clinical performance of the denture bases and their longevity.

**Supplementary Information:**

The online version contains supplementary material available at 10.1186/s12903-025-06142-8.

## Background

Removable prosthetic restorations are still considered the gold standard treatment for edentulous patients as they are crucial for maintaining a healthy lifestyle by restoring esthetics, masticatory functions, occlusion, and phonetics [1, 2]. The conventional heat-curing methods used for fabricating removable restorations can produce dentures that have satisfactory aesthetic and functional results. However, these conventional techniques require several laboratory procedures to fabricate the removable restorations. Additionally, the restorations fabricated by these methods can undergo significant volumetric shrinkage, color changes, and deterioration of mechanical properties after being exposed to oral cavity conditions. This leads to dissatisfaction of the patient due to discomfort during mastication and inferior aesthetics over time [3]. Digital dentistry has been introduced to overcome the drawbacks of conventional fabrication techniques. Recently, 3D-printers are used to fabricate a variety of dental prostheses, including dentures because of its superior accuracy and precision. 3D printing, or Additive Manufacturing (AM) technique includes model designing using specific software, which is saved as a standard tessellation language (STL) file, followed by model slicing to create a g-code. After feeding the g-code into the 3D printer, the denture is printed using dental resin materials like photo-polymerized polymethylmethacrylate (PMMA) denture base resins. 3D-printing also provides a rapid and efficient process of fabrication with minimal material waste [2, 4, 5]. However, 3D-printed denture base resins may have limited mechanical properties such as low flexural strength and low elastic modulus [6, 7]; therefore, several attempts have been made to improve the mechanical, biological, and physical properties of 3D-printed denture base resins [8, 9]. Recently, the use of nanocomposites, which are materials that are composed of inorganic nanoparticles evenly distributed within organic polymer matrices, has been the focus of numerous research studies. These nanocomposites were found to have superior performance and distinctive features compared to traditional 3D-printed denture base materials [10]. Numerous studies have explored how incorporating fillers such as metal-oxide nanoparticles (zirconium dioxide, titanium oxide) [11, 12], carbon, and nanoglass [13] can improve the performance of 3D-printed denture base resins.

Nano-clay is one of the most commonly used nanoparticles that have been successfully integrated into the polymethyl methacrylate denture base matrix. The incorporation of such particles, which have high surface area, in the matrix can result in significant enhancements of surface and mechanical properties of clay-polymer composites [14, 15]. Zeolite nanoparticles are crystalline porous aluminosilicate nano-clay biomaterials that consist of a tetrahedral arrangement of silicon cations (Si^4+^) and aluminum cations (Al^3+^) that are surrounded by four oxygen anions (O^2−^) [16]. The unique structure and porous nature of zeolites offer a diverse array of functionalities which make zeolites promising materials for dental applications. Zeolites have been found to have ion exchange properties and absorption capacity, enabling them to trap and bind with antimicrobial metal ions, as well as water and organic molecules, and exchange them with other molecules from the surrounding environment. Also, zeolites possess high thermal and chemical stability as well as excellent biocompatibility which can improve the mechanical, physical, and biological properties of denture base materials [17, 18].

Previous research has shown that reinforcing resins with nano-clay particles can enhance the mechanical characteristics of the reinforced polymers and prevent crack propagation [19]. Research has also explored the effects of nano-clay reinforcements on the properties of heat-cured denture base resins [20] as well as the modification of PMMA with nano-clay particles [21]. Additionally, 3D printed dental composites of acrylic resin and nano-clay were discussed before [22]. However, modifying 3D-printed denture resins with specific zeolite nano-clay particles has not been previously discussed.

To ensure the effective performance of removable prostheses, it is crucial to enhance their properties, while also rigorously testing these improvements to meet functional and patient satisfaction standards. Fracture toughness which is the maximum stress a material can endure before crack initiation and propagation is one critical property [23]. Equally important is flexural strength, which assesses a resin's ability to withstand the complex stresses of mastication, including compressive, tensile, and shear forces [24]. Dimensional accuracy also significantly impacts long-term success, as precise fit ensures proper retention of the denture, efficient chewing, and clear speech [6, 25]. Additionally, surface roughness must be minimized, as rough denture bases can stain easily and promote microbial adhesion, increasing the risk of oral infections like denture stomatitis. A smooth surface is therefore vital for both hygiene and patient comfort [26].

The aim of this study was to evaluate the effect of modifying a 3D-printed denture base resin with nanozeolite (NZ) particles on dimensional accuracy (DA), fracture toughness (FT), biaxial flexural strength (BFS), and surface roughness (SR) of these printed resins as well as assess the effect of thermal cycling on the previously mentioned properties. The null hypothesis proposed that the difference in nanozeolite content and thermal cycling would not affect the mechanical properties and surface roughness of the 3D-printed denture base resins.

## Methods

### Sample size

Based upon the assumptions of a 95% level of confidence and an 80% study power as well as a pilot study conducted along with data reported in previous literature, the total sample size was calculated to be 240 [13, 23, 27, 28]. A computer program (G*power 3.0.10; Heinrich Heine University Düsseldorf) [29] and Rosner technique [30] were used for sample size calculation.

### Preparation of reinforced resin groups and designing of specimens

NZ particles (45 ± 40-nm average diameter; Nano Gate Co., Egypt) were added to a methacrylate-based 3D-printed denture base resin (Denture Base LP; Formlabs, USA) to formulate the following groups: Control group (with no NZ addition), 0.25 wt% NZ-modified resin, and 0.5 wt% NZ-modified resin. The modified resin groups were put in glass beakers and stirred using a magnetic stirrer (F91 T; Falc, Italy) at 500 rpm for 1 h, then the beakers were put in an ultrasonic bath (T-14; L & R manufacturer, USA) for another 1 h for improving the dispersion of nanofillers before printing [31].

The denture base specimens were designed as standard tessellation language (STL) files with a computer-aided design (CAD) software program (Meshmixer 3.5, Autodesk Inc., USA). FT specimens (*n* = 72) were designed according to ISO 20795–1:2013 as rectangular blocks (39 mm × 8 mm × 4 mm) with a 3-mm notch [23]. The specimens for the BFS test were designed in the form of 12 × 2-mm disks (*n* = 60) [28]. The SR specimens were designed as 10 × 2-mm disks (*n* = 108) [32]. After the specimens’ designing, the STL files were transferred to a 3D-printer (Form 2, Formlabs, USA) software, and the specimens were then obtained by printing at 45° for maximum printing accuracy with a layer thickness of 50 µm [33]. Afterward, post-polymerization of the printed specimens was conducted following the instructions of the manufacturer. The SR specimens were finished and polished prior to testing according to El-Din et al. and Alfouzan et al. [34, 35].

Then, half of the specimens of each test -except for dimensional accuracy- from each group were thermo-cycled for 600 cycles between 5 °C and 55 °C, representing 6 months of intraoral usage of the prosthesis, while the other half was tested without being aged [23, 36].

### Dimensional accuracy

To assess the printing DA, the FT specimens’ dimensions were measured after printing and postprocessing prior to testing (*n* = 54). The specimens’ dimensional accuracy was estimated by comparing the measured dimensions of printed specimens to the STL file dimensions of the specimens. The length, thickness, and width of each printed specimen were measured using a digital calliper (01407 A, Neiko tools, China) with a 0.01 mm precision. Each dimension was measured three times (at the specimen’s centre and at a 1-mm distance away from the specimen edge). After that, the average measurement was obtained for each specimen. For each dimension, the accuracy was evaluated by calculating the percent error following the formula: A = ((MD- RD)/RD) × 100 where A is the accuracy which corresponds to the dimensional percent error, MD is the measured dimension, and RD is the virtual reference dimension [27].

### Fracture toughness, work of failure, and Weibull modulus

A sharp razor blade was used to make a cut at the bottom of the notch (0.1–0.4 mm) of each specimen. After that, a 3-point bending setup was used over which the notched specimens were supported and the fracture load in Newton was measured using a universal testing machine (UTM) (5ST, Tinius Olsen, England) at 1 mm/min. K_IC_ was calculated using the following equation:

$${K}_{IC} = \left(\left(f.Pmax.lt\right) / \left({bt.ht}^{3/2}\right)\right) \sqrt{{10}^{-3}}$$

with *K*_*IC*_: fracture toughness (*MPa.m*^1*/*2^); *Pmax*: fracture load (*N*); *lt*: span (*mm*); *bt*: specimen width (*mm*); *ht*: specimen height (*mm*); *f*: a geometric function of *x*. *f* (*x*) = 3*x*^1*/*2^ (1*.*99—*x*(1—*x*)(2*.*15—3*.*93*x* + 2*.*7*x*^2^)) (2(1 + 2*x*)(1—*x*)^3*/*2^) and *x* = *a/ht* with *a*: sum of notch depth (*mm*) and cut depth (*mm*) [23]. After the test, the fractured surfaces were investigated using a scanning electron microscope (SEM) (JSM-IT200, JEOL, USA). The work of failure (WOF) *(J/m*^*2*^*)* was calculated from the integral area (U) (*N.mm*) of the load–deflection curve [37]:

$$WOF = \left(U/ \left(2bt \left(ht -a\right)\right)\right) \times 1000$$

The Weibull distribution, Weibull modulus (m), and the characteristic fracture toughness (K_0_) were estimated at 95% CI using Origin software (OriginPro, Version 2024, OriginLab Corporation, USA) [38].

### Biaxial flexural strength

For the BFS assessment, the disks were centrally positioned onto an O ring with a 6-mm diameter knife-edged ring support on the UTM. Then, load was applied with a 1 mm/min crosshead speed by a 1-mm ball-tipped piston to the disks’ centre. Following that, the BFS was calculated using the equation: BFS = (P/h^2^)[0.606 log_e_ (a/w) + 1.13], where P is the ultimate load, a is the O ring knife-edge support radius, and h is the disk thickness [28]. Afterward, the specimens’ fracture pattern was evaluated with a stereomicroscope (× 18) (Olympus B061, Olympus Optical Co. Ltd, Japan).

### Surface roughness assessment

The average SR values (µm) were measured on the 3D-printed disks using a contact profilometer (Marsurf PS10; Mahr GmbH) [13]. SEM analysis was performed for the surface of representative specimens from each group.

### Statistical analysis

The results were analysed using statistical software (IBM SPSS Statistics, v.27.0, IBM, USA). The normality check was conducted for all variables using the Shapiro–Wilk test and Q-Q plots. The data for FT, WOF, BFS, and SR were normally distributed and hence they were analysed using the two-way ANOVA test followed by Tukey post hoc test to evaluate the effect of nanofiller content and thermal cycling. DA and percentage change data were non-normally distributed and therefore they were analysed with the Kruskal–Wallis test succeeded by Dunn post hoc test with Bonferroni correction. The specimens’ failure predictability and lifespan expectancy were assessed using Weibull analysis. Percentage change for all tested variables was calculated using the formulation ([values after thermal cycling—values before thermal cycling]/values before thermal cycling) × 100. The significance level was adjusted to *P* ≤ 0.05.

## Results

### Dimensional accuracy

The printing DA results displayed in Table 1 show that the control revealed the highest percent error in length and width (−0.67% and −0.19% respectively) with no significant difference among the study groups, whereas the 0.5% NZ group presented the highest percent error in thickness (−1.50%). The only statistically significant difference was observed between the control and 0.5% NZ group (*P* = 0.027) regarding the thickness dimension only.

**Table 1:** Comparison of length, width, and thickness percent error (%) between the study groups

		Control	0.25% Nanozeolite	0.5% Nanozeolite	*P*
Length	Median (IQR)	−0.67 (1.24)	−0.08 (1.31)	−0.31 (1.18)	0.451
	Min	−1.21	−1.13	−1.03	
	Max	0.03	0.18	0.15	
Width	Median (IQR)	−0.19 (4.38)	−0.06 (6.13)	−0.12 (3.63)	0.672
	Min	−1.63	−2.00	−2.13	
	Max	2.75	4.13	1.50	
Thickness	Median (IQR)	−0.63 (3.75)	−1.25 (5.25)	−1.50 (5.50)	0.031*
	Min	−3.25	−2.75	−5.75	
	Max	0.50	2.50	−0.25	
	*Thickness pairwise comparison*		*P*_*1*_ = 0.870	*P*_*2*_ = 0.027*	*P*_*3*_ = 0.361

*IQR* interquartile range, *Mdn* median, *SD* standard deviation, *x̄* mean

*P*_1_: Comparison between control and 0.25% Nanozeolite, *P*_2_: Comparison between control and 0.5% Nanozeolite, *P*_3_: Comparison between 0.25% Nanozeolite and 0.5% Nanozeolite

^*^Statistically significant difference (*P* ≤ 0.05)

### Fracture toughness, work of failure, and Weibull modulus

The two-way ANOVA (Table 2) displayed a significant statistical interaction between the resin filler content and thermal cycling on the FT results (*P* = 0.001, F = 7.501, Ƞp^2^ = 0.185) with the thermal aging displaying the uppermost effect (Ƞp^2^ = 0.640). Although the control group recorded the highest FT values before thermal cycling (0.66 ± 0.06 MPa.m^1/2^), it revealed the highest percentage decrease in FT after aging (−34.30%). The 0.5% NZ group recorded the highest mean value after aging (0.48 ± 0.04 MPa.m^1/2^) with the least percentage decrease (−19.85%) after thermal cycling (Table 3).

**Table 2:** Two-way ANOVA for effects of zeolite nanofiller content and thermal cycling and their interaction on fracture toughness, work of failure, biaxial flexural strength, and surface roughness

	Variables	Mean square	F test	*P*	Ƞp^2^	R^2^_adj_
Fracture toughness	Nanofiller content	0.121	41.005	< 0.001*	0.554	
	Thermal cycling	0.344	117.154	< 0.001*	0.640	
	Interaction	0.022	7.501	0.001*	0.185	
	Corrected model	0.126	42.833	< 0.001*	0.764	0.747
Work of failure	Nanofiller content	39434.226	36.823	< 0.001*	0.527	
	Thermal cycling	249582.900	233.056	< 0.001*	0.779	
	Interaction	6818.013	6.367	0.003*	0.162	
	Corrected model	68417.476	63.887	< 0.001*	0.829	0.816
Biaxial flexural strength	Nanofiller content	742.072	19.234	< 0.001*	0.416	
	Thermal cycling	85.610	2.219	0.142	0.039	
	Interaction	4.635	0.120	0.887	0.004	
	Corrected model	315.805	8.186	< 0.001*	0.431	0.378
Surface roughness	Nanofiller content	0.004	19.904	< 0.001*	0.281	
	Thermal cycling	0.000	1.298	0.257	0.013	
	Interaction	0.002	8.386	< 0.001*	0.141	
	Corrected model	0.003	11.575	< 0.001*	0.362	0.331

*Ƞp*^*2*^ partial eta squared, *R*^*2*^_*adj*_ adjusted R squared

^*^Statistically significant difference (*P* ≤ 0.05)

**Table 3:** Descriptive values of fracture toughness (MPa.m^1/2^), work of failure (J/m^2^), biaxial flexural strength (MPa), and surface roughness (μm), of study groups before and after thermal cycling

	Thermal cycling	Control	0.25% Nanozeolite	0.5% Nanozeolite
Fracture toughness (*n* = 12)	Before: x̄ (SD)	0.66 (0.06)	0.50 (0.07)	0.55 (0.03)
	After: x̄ (SD)	0.47 (0.07)	0.35 (0.04)	0.48 (0.04)
	% Change: Mdn (IQR)	−34.30 (29.80)	−28.95 (47.20)	−19.85 (32.90)
Work of failure (*n* = 12)	Before: x̄ (SD)	277.49 (57.81)	179.28 (36.85)	242.27 (30.48)
	After: x̄ (SD)	121.29 (20.94)	75.48 (13.80)	149.00 (12.92)
	% Change: Mdn (IQR)	−59.15 (48.70)	−57.60 (11.40)	−39.80 (34.30)
Biaxial flexural strength (*n*=10)	Before: x̄ (SD)	34.65 (2.78)	45.87 (6.53)	41.24 (12.05)
	After: x̄ (SD)	31.47 (1.77)	44.55 (2.14)	38.57 (5.32)
	% Change: Mdn (IQR)	−7.20 (30.80)	−1.10 (44.90)	−3.00 (99.70)
Surface roughness (*n* = 18)	Before: x̄ (SD)	0.065 (0.02)	0.065 (0.01)	0.055 (0.02)
	After: x̄ (SD)	0.085 (0.01)	0.058 (0.01)	0.051 (0.01)
	% Change: Mdn (IQR)	48.15 (94.70)	−10.00 (22.80)	−6.55 (38.10)

*IQR* interquartile range, *Mdn* median, *SD* standard deviation, *x̄* mean

Regarding WOF, the results in Table 2 revealed an inclusive significant interaction between the filler content and aging (*P* = 0.003, F = 6.367, Ƞp^2^ = 0.162) with the thermal cycling presenting the topmost effect (Ƞp^2^ = 0.779). The control revealed the highest value (277.49 ± 57.81 J/m^2^) before thermal cycling, whereases the 0.5% NZ group recorded the highest value (149.00 ± 12.92 J/m^2^) with the least percent decrease (−39.80%) after aging (Table 3).

**Fig. 1:**
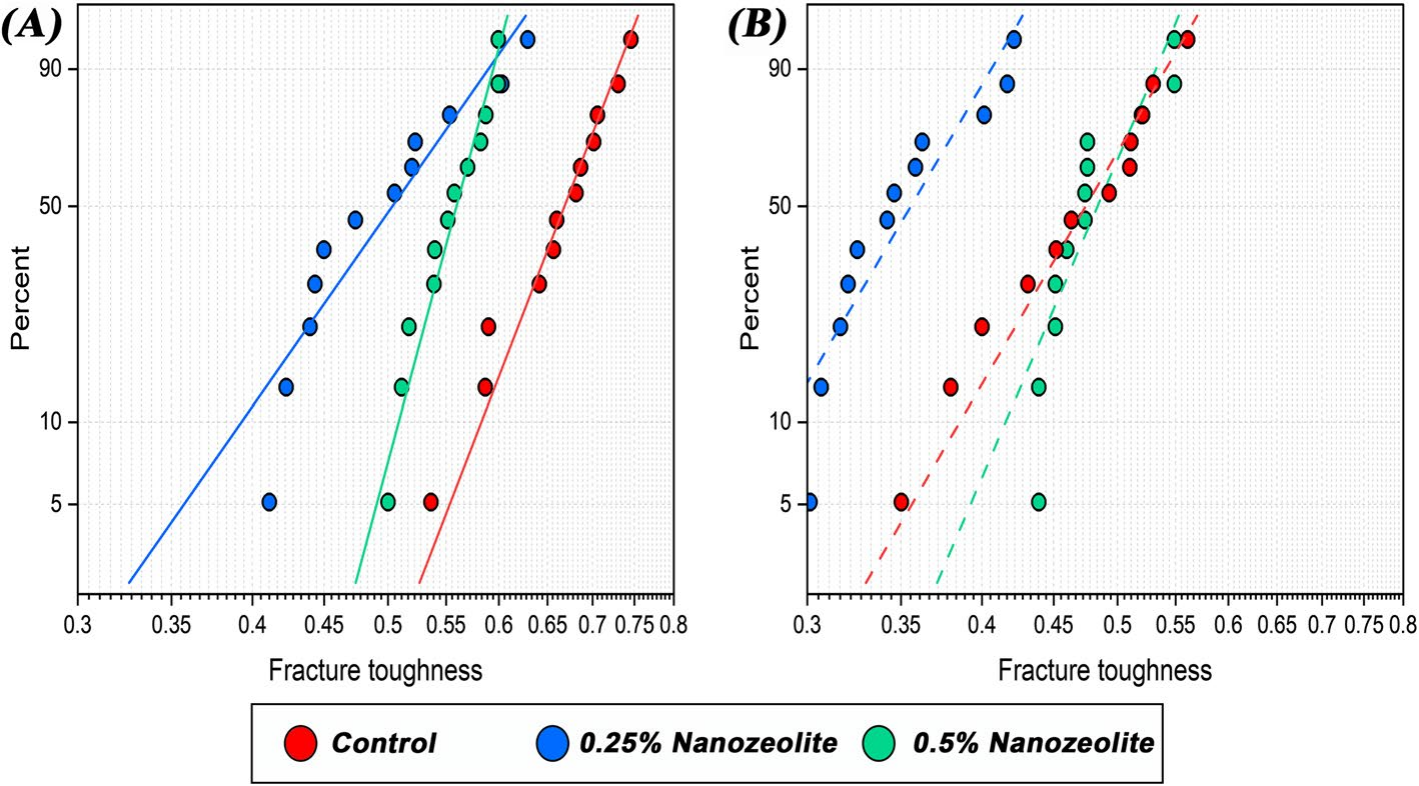
Weibull modulus of fracture toughness of study groups. **A** Before thermal cycling. **B** After thermal cycling

The results of the Weibull distribution analysis are displayed in Fig. 1 and Table 4. Before thermal cycling, all the study groups demonstrated superior characteristic fracture toughness (K_0_) compared to their counterparts subjected to thermal aging. The 0.5% NZ group exhibited the highest modulus values (*m*) both before and after thermal aging (Table 4).

**Table 4:** Weibull statistics for the study groups

Group		m (Weibull shape)				K_0_ (Weibull scale)			
		Estimate	SD error	Lower	Upper	Estimate	SD error	Lower	Upper
Before thermal cycling	Control	13.7961	3.2156	8.7369	21.7850	0.6864	0.0151	0.6574	0.7166
	0.25% Nanozeolite	7.5892	1.6333	4.9775	11.5714	0.5284	0.0214	0.4882	0.5720
	0.5% Nanozeolite	19.8558	4.5638	12.6543	31.1554	0.5699	0.0088	0.5530	0.5873
Afterthermal cycling	Control	9.0922	2.1327	5.7413	14.3989	0.4939	0.0165	0.4626	0.5273
	0.25% Nanozeolite	9.0560	1.9679	5.9152	13.8644	0.3704	0.0126	0.3466	0.3958
	0.5% Nanozeolite	12.4600	2.6330	8.2346	18.8536	0.4981	0.0123	0.4746	0.5228

*SD* standard deviation

**Fig. 2:**
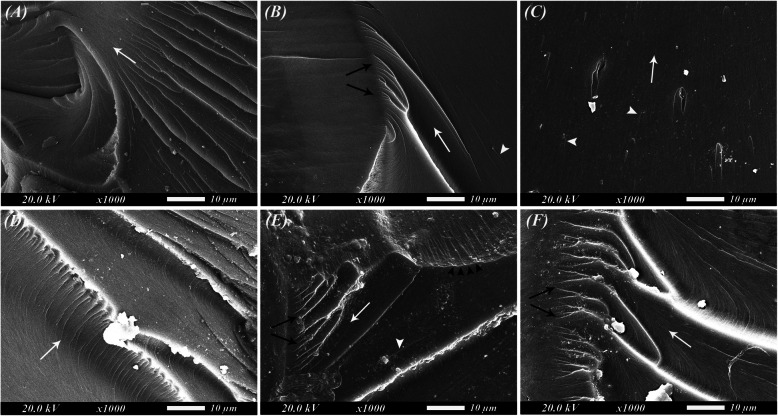
SEM analysis of fractured specimens after fracture toughness testing (original magnification × 1000). **A-C** SEM images before thermal cycling. **D-F** SEM images after thermal cycling. **A** and **D** Control. **B** and **E** 0.25% nanozeolite. **C** and **F** 0.5% nanozeolite. White arrow indicating direction of crack propagation. Black arrows denoting twist hackles. Black arrowheads denoting an arrest line. White arrowheads denoting wake hackles

SEM analysis of the fractured specimens was carried out after FT testing before and after thermal cycling. Upon observing the SEM images before aging (Fig. 2), some hackle lines were seen in the control group (Fig. 2A). For the specimens that were reinforced with 0.25% NZ particles (Fig. 2B), smooth mirror-like surfaces with some twist hackles and wake hackles could be observed indicating brittle failure. SEM images of the 0.5% NZ specimens (Fig. 2C) revealed the presence of some clusters with the presence of some cracking and wake hackles. After thermal cycling, SEM images (Fig. 2D-F) displayed the presence of many irregularities and twist hackles compared to those seen in the images before aging. Moreover, detached protruding particles could be seen in those images. An arrest line could be observed in the representative SEM image of the 0.25% NZ group (Fig. 2E).

### Biaxial flexural strength

Regarding the BFS, the results of two-way ANOVA (Table 2) revealed a significant effect for the filler content (*P* < 0.001, F = 19.234, Ƞp^2^ = 0.416) on BFS, with the 0.25% NZ group displaying the highest mean values before (45.87 ± 6.53 MPa) and after thermal cycling (44.55 ± 2.14 MPa) and the least percent decrease after aging (−1.10%) (Table 3).

### Surface roughness

**Fig. 3:**
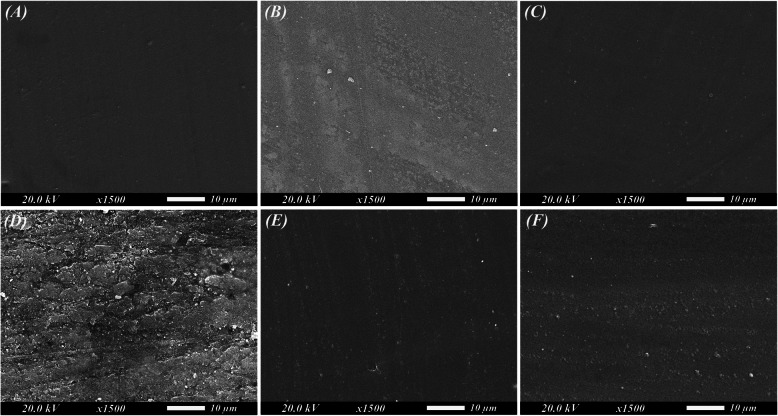
SEM images for polished surface of roughness specimens (original magnification × 1500). **A-C** SEM images before thermal cycling. **D-F** SEM images after thermal cycling. **A** and** D** Control. **B** and** E** 0.25% nanozeolite. **C** and** F** 0.5% nanozeolite

Regarding the SR results, the two-way ANOVA (Table 2) revealed a statistically significant interaction between the filler content and thermal aging (*P* < 0.001, F = 8.386, Ƞp^2^ = 0.141) with the filler content presenting the uppermost effect (Ƞp^2^ = 0.281). The 0.5% NZ group displayed the lowest SR mean values before (0.055 ± 0.02 μm) and after thermal aging (0.051 ± 0.01 μm). After aging, the control group revealed a percentage increase (48.15%) while the 0.25% NZ and 0.5% NZ groups presented a percentage decrease of −10.00% and −6.55% respectively (Table 3). This could also be verified by the SEM images (Fig. 3) which showed that the specimens had smooth surfaces with no apparent gaps or voids. However, after aging, the control appeared to have a rougher surface than before aging (Fig. 3D) which aligns with the results of the SR mentioned above.

Pairwise comparisons regarding FT, WOF, BFS, SR, and percent change among groups are shown in Table 5, and Supplemental Table 1.

**Table 5:** Pairwise comparisons among groups regarding fracture toughness, work of failure, biaxial flexural strength, and surface roughness

Groups	Compared with	*P*			
		Fracture toughness	Work of failure	Biaxial flexural strength	Surface roughness
Control	0.25% Nanozeolite	< 0.001*	< 0.001*	< 0.001*	< 0.001*
	0.5% Nanozeolite	0.013*	1.000	0.003*	< 0.001*
0.25% Nanozeolite	0.5% Nanozeolite	< 0.001*	< 0.001*	0.027*	0.045

^*^Statistically significant difference (*P* ≤ 0.05)

## Discussion

Denture bases should be well adapted to their underlying tissues to attain high retention and stability thus achieving patient comfort and avoiding supporting soft tissue traumatic ulceration. Therefore, maximizing the DA of such prostheses is of great importance [25, 39]. For stereolithography (SLA)-printed bases, dimensional discrepancies could arise from a combination of factors including the printer and resin type, printed layer thickness, building orientation, light intensity, and post-curing process [6]. The printing orientation could influence DA owing to variations in reproducibility across the X, Y, and Z axes [40]. Studies that investigated the effect of printing orientation on DA are sparse with conflicting results. Furthermore, to the authors’ knowledge, no previous studies have investigated the influence of nanoparticle addition on the DA of printed denture bases. The results of the present study displayed an improvement regarding the accuracy of length and width dimensions in the reinforced groups but with no significant difference compared to the control (Table 1). This slight enhancement could be attributed to the reduction of polymerization shrinkage, which accompanies the formation of covalent bonds reducing the distance between monomer molecules [41], by decreasing the shrinkable monomer fraction with the addition of zeolite nanofiller which filled the gaps in between the resin chains [14, 42, 43]. The 0.5% nanozeolite group displayed lower DA regarding length and width compared to the 0.25% NZ group which could be attributed to nanofiller agglomeration with increasing filler content [19]. These nanofiller clusters could affect printing accuracy by scattering light during printing [44]. Conversely, the thickness percent error increased with the addition of zeolite nanofillers compared to the control which could be assigned to the anisotropy of the printed parts [27] besides the stepwise layer linking during printing with the 45° orientation which produces edged steps between the printed layers [40].

**Fig. 4:**
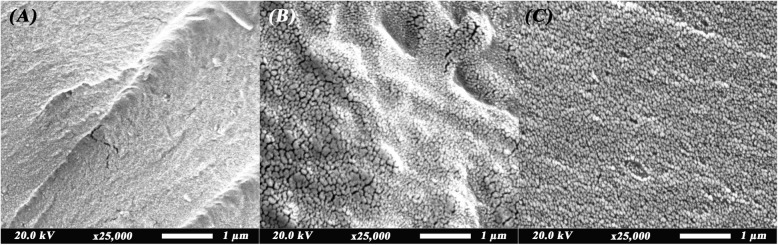
SEM micrographs (original magnification × 25,000) showing microstructure of the resin in **A**, control group and dispersion of nanozeolite in resin in **B**, 0.25% nanozeolite and **C**, 0.5% nanozeolite

Fracture toughness represents the ability of the material to resist crack propagation [23] and it is affected by the amount of nanofillers, filler dispersion, and filler-matrix interaction [21]. In this study, the small size of nanozeolite particles used might have generated some microcracks (Fig. 4B) that could have caused the decrease in fracture toughness at 0.25% filler loading. Since the nanoparticles in our study are non-silanized, the weakness of the resin-filler interface led to lack of stress transfer to the filler in addition to stress concentration resulting in a decrease of fracture toughness [21]. The agglomeration of the nanoparticles at 0.5% is generally considered a drawback, however, this agglomeration might have led to the presence of larger sized particles (Fig. 4C) leading to crack deflection or bridging thus regaining the fracture toughness to be closer to that of the control group. The fracture toughness might have benefited from the high aspect ratio of nanozeolite which resulted in proper dispersion of stress with higher filler loading [45]. Although the addition of nanozeolite has decreased the fracture toughness compared to control, this addition improved the resistance to the deterioration of fracture toughness after aging. The aging process imparts a plasticizing effect on the resin chains leading to their separation and weakening. In addition to this, an increase in water sorption and the hydrolytic effect of water lead to a decrease in FT [37]. Since nanozeolite is known for its thermal stability and resistance to thermal changes, the resin chain separation and water sorption have decreased with the addition of nanozeolite thus improving the FT with increasing the filler loading [46]. The results of this study coincide with those obtained by Alhareb et al. who added 7.5% nitril rubber along with alumina and zirconia particles to heat cured denture base resins. They found that the addition of 2.5% of each filler type increased the fracture toughness significantly compared to control group [47]. The optimum filler concentration differs according to the type of filler used as deducted by Alhotan et al. After the addition of zirconia or titania particles, the fracture toughness increased compared to control but only up to 3%, then started decreasing with higher filler loading. On the contrary, low filler percentages of E-glass fibre did not improve the fracture toughness, while 7% of said filler increased the fracture toughness significantly [12].

The fracture features seen in SEM micrographs (Fig. 2) express features of brittle failure [10, 48]. This can be attributed to the ceramic nature of the reinforcing particles as nanozeolites are made up of aluminosilicate building blocks [49]. The arrest line seen in the representative SEM images of the 0.25% NZ (Fig. 2E) proves the ability of the reinforcing particles to stop crack propagation and enhance the mechanical properties of the 3D-printed denture base material. The effect of nanozeolite is also reflected in the Weibull modulus, where the 0.5% group showed the highest stability and best failure predictability both before and after aging indicating a consistency in the failure pattern. Nanozeolite particles possess a toughening effect due to intercalation and exfoliation which bind the resin chains restricting their separation and movement despite heating and aging [22]. Such an effect required higher energy to cause delamination and fracture of the specimens with higher nanozeolite loading (0.5%), especially after thermocycling which is indicated by the higher WOF of this group surpassing that of control after aging [50].

Denture bases require high bending resistance to resist the repeated flexing stresses applied during the different oral functions [24, 51]. The BFS test was chosen in the current study as it provides a better indicator of specimens'strength compared to the uniaxial flexural test owing to the position of the specimen over the supports, where the edges of the specimens, in which processing flaws might be present, are exposed to lower stress concentration during testing [52]. The results revealed a significant improvement in BFS in the nanozeolite-reinforced resin groups. This enhancement could be assigned to proper dispersion of zeolite nanoparticles within the resin matrix providing intercalated or exfoliated morphology filling the voids and gaps in the polymeric matrix thus restricting the chains'movement [10, 53], besides the greater rigidity of the fillers compared to the softer matrix [20] which improved the BFS. Furthermore, the small size of the used nanoparticles (45 ± 40-nm) furnishes a great surface area improving the interfacial interaction between the resin and fillers [19, 20] allowing transfer of the bending stresses to the stronger fillers, providing better distribution of these stresses throughout the resin, thus enhancing the BFS [51]. The 0.25% NZ group displayed higher BFS compared to the 0.5% NZ one before and after aging. This effect could have resulted from the agglomeration tendency of nanoparticles with increasing the filler content providing better results for the group with lower filler concentration (0.25% NZ) [19].

After thermal cycling, all study groups showed a reduction in BFS average values (Table 3). The resultant deterioration could be caused by the plasticization of the polymer matrix during thermal cycling under the influence of water [32]. Furthermore, temperature fluctuations can accelerate water diffusion through the matrix, causing the polymer chains to be pulled apart and generating internal stresses with subsequent cracking [26, 32, 54]. Nonetheless, the 0.25% NZ group revealed the least percentage decrease after thermal cycling (Table 3). This could be assigned to the more favorable uniform nanofiller distribution with small concentrations [19, 55]. To the best of the authors'knowledge, no prior research has investigated the impact of incorporating nanozeolite into 3D-printed denture base resins on their BFS. Consequently, the study's findings could not be perfectly compared to those of others. However, these findings agreed with Joseph et al. who reported higher uniaxial flexural strength for heat-cured resin groups reinforced with lower concentration of montmorillonite nano-clay [20]. The results are also in line with those reported by Alzayyat et al. where the heat-cured resin modified with 0.25% SiO_2_ presented higher flexural strength compared to the 0.5% SiO_2_ group [56].

**Fig. 5:**
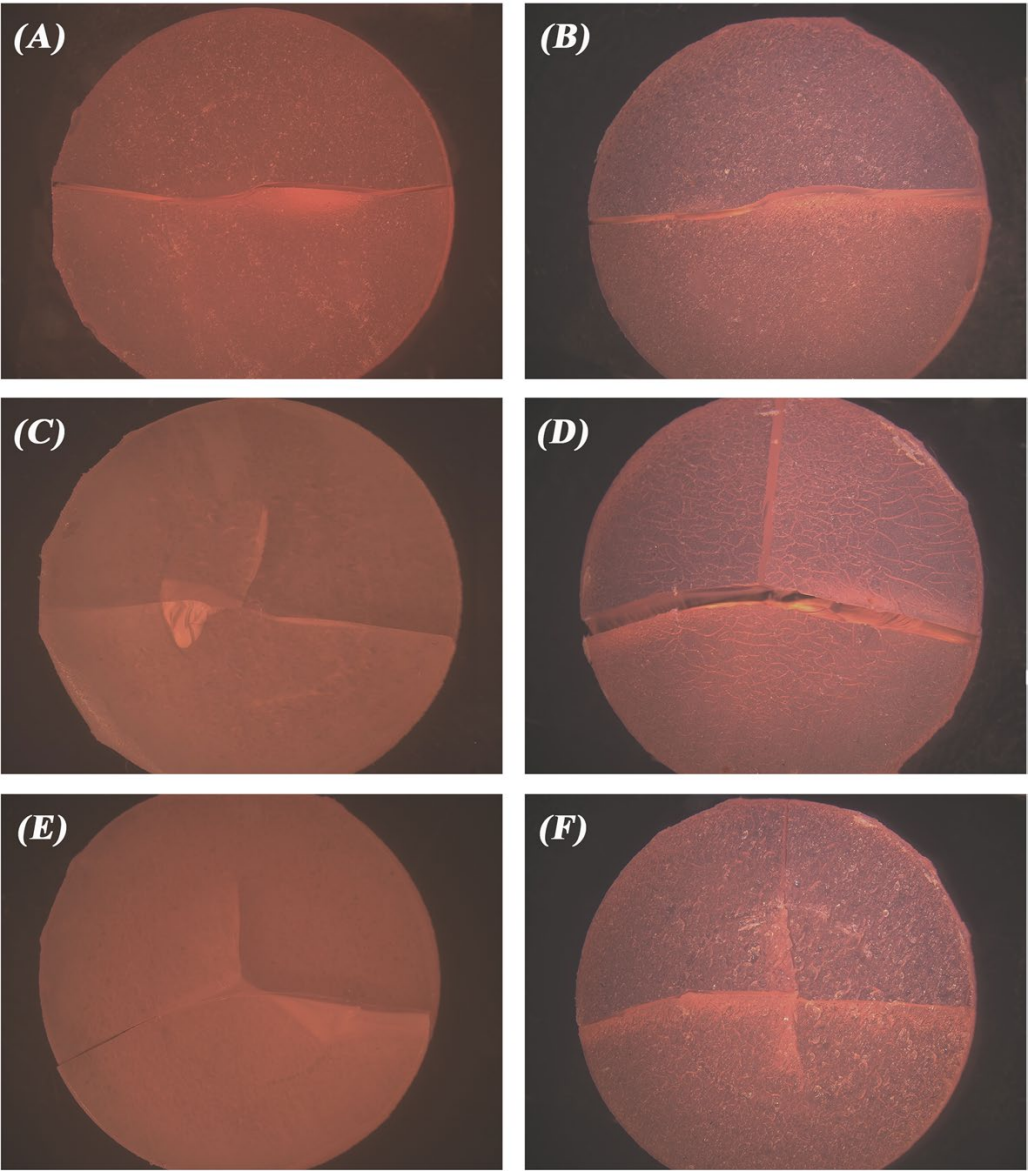
Representative stereomicroscopic images (original magnification × 18) of fractured specimens following biaxial flexural strength in each group. **A** Control before thermal cycling. **B** Control after thermal cycling. **C** 0.25% nanozeolite before thermal cycling. **D** 0.25% nanozeolite after thermal cycling. **E** 0.5% nanozeolite before thermal cycling. **F** 0.5% nanozeolite after thermal cycling

The investigation of the broken BFS specimens showed that the control group’s specimens broke into 2 pieces. On the other hand, the majority of the 0.25% and 0.5% NZ groups’ specimens were broken into 3 pieces or more (Fig. 5). This could be attributed to the uniform dispersion of zeolite nanoparticles throughout the polymeric matrix causing crack diversion in multiple directions during propagation [28]. Moreover, the cracks in the control group passed the specimen from edge to edge, while in nanozeolite-reinforced groups, some cracks were arrested and did not reach the edge of the specimen (Fig. 5C, E). This could be assigned to the interlocking of the nanofillers within the resin matrix which broke up the propagation of some cracks providing a toughening mechanism [14, 28].

Regarding the SR result, increasing the concentration of zeolite nanoparticles in the resin improved the surface properties, with the 0.5% NZ group displaying the lowest SR mean values before and after thermal cycling. The explanation for the decreased SR could be due to proper dispersion and interaction between the zeolite nanoparticles and polymer chains via physical and chemical cross-linking. These interactions increase the packing density of particles in 3D-printed resin, which increases their surface smoothness. Furthermore, the higher proportion of nanozeolite in 0.5% group is associated with the probability of nanoparticle accumulation increasing in the resin core while, lower concentration on the surface thus improving the surface properties [57, 58]. Additionally, the water absorption resistance, which is primarily improved by the high concentration of hydrophobic zeolite nanoparticles that give the polymer composite colloidal stability, is responsible for the notable decrease in SR in the 0.5% NZ group after aging [59]. Achieving lower surface roughness with increased filler loading is not a common occurrence as most fillers tend to agglomerate, protrude from the surface, and separate resin chains, thus increasing surface roughness. Fatalla et al. reported that the addition of silica and zirconia increased surface roughness of denture base materials compared to control [51]. The addition of nano silica was also a contributor of increased surface roughness of denture base materials and the effect was aggravated by thermocycling according to Gad et al. [26].

The limitations of this study include using only one type of denture base resin, one type of filler, and only one 3D printing technique. Also, longer thermocycling durations need to be employed to have a more accurate expectation of the intraoral performance of the prosthesis. This study is an in-vitro study which focused on mechanical and surface properties of the nanozeolite filled resin. Future studies should be directed towards evaluating biocompatibility, antibacterial effect, wear resistance, and color stability to evaluate the potential of this reinforced resin for clinical use.

## Conclusions

Based on the results of this study, it can be concluded that adding zeolite nanoparticles as a reinforcing phase could improve the mechanical properties and surface roughness of 3D-printed denture base resins and mitigate the negative effects that thermocycling can have on the properties and performance of these resins. However, care should be given to the optimum percentage added to the resin to attain optimum properties which would enhance the clinical performance of the denture bases and their longevity.

## Supplementary Information

Supplementary Material 1.

## Data Availability

The data that support the findings of this study are available from the corresponding author upon reasonable request.

## Abbreviations

3D: Three-dimensionally
AM: Additive manufacturing
PMMA: Polymethylmethacrylate
NZ: Nanozeolite
DA: Dimensional accuracy
FT: Fracture toughness
WOF: Work of failure
BFS: Biaxial flexural strength
SR: Surface roughness
SENB: Single edge notched beam
STL: Standard tessellation language
CAD: Computer-aided design
MD: Measured dimension
RD: Virtual reference dimension
UTM: Universal testing machine
SEM: Scanning electron microscope
