# Supplementary material for: Assessment of dimensional accuracy, fracture toughness, biaxial flexural strength, and surface roughness of nanozeolite reinforced 3D-printed denture base resin (In-vitro study)
Source: BMC Oral Health. 2025 May 21;25:754. doi: 10.1186/s12903-025-06142-8 (PMC12096564; doi:10.1186/s12903-025-06142-8)
Supplement: Supplementary file 1 — Supplementary Material 1. [file 12903_2025_6142_MOESM1_ESM.docx]

**Supplemental Table 1**. Pairwise comparisons among groups regarding percent change of fracture toughness, work of failure, biaxial flexural strength, and surface roughness before and after thermal cycling.

| Groups | Compared with | *P* | | |
| --- | --- | --- | --- | --- |
|  |  | Fracture toughness | Work of failure | Surface roughness |
| Control | 0.25% Nanozeolite | 1.000 | 1.000 | 0.000* |
|  | 0.5% Nanozeolite | 0.050* | 0.004* | 0.000* |
| 0.25% Nanozeolite | 0.5% Nanozeolite | 0.171 | 0.002* | 1.000 |

*Statistically significant difference (*P*≤0.05).
